# Congenital Cataracts and Gut Dysmotility in a *DYNC1H1* Dyneinopathy Patient

**DOI:** 10.3390/genes7100085

**Published:** 2016-10-14

**Authors:** Rose Gelineau-Morel, Marshall Lukacs, K. Nicole Weaver, Robert B. Hufnagel, Donald L. Gilbert, Rolf W. Stottmann

**Affiliations:** 1 Division of Neurology, Cincinnati Children’s Hospital Medical Center, Cincinnati, OH 45229, USA; Rose.Gelineau.Morel@cchmc.org (R.G.-M.); Donald.Gilbert@cchmc.org (D.L.G.); 2 Division of Human Genetics, Cincinnati Children’s Hospital Medical Center, Cincinnati, OH 45229, USA; Marshall.Lukacs@cchmc.org (M.L.); Kathryn.Weaver@cchmc.org (K.N.W.); Robert.hufnagel@nih.gov (R.B.H.); 3 Division of Developmental Biology, Cincinnati Children’s Hospital Medical Center, Cincinnati, OH 45229, USA

**Keywords:** dynein, polymicrogyria, cortical development, cataracts, gut dysmotility

## Abstract

Whole exome sequencing continues to end the diagnostic odyssey for a number of patients and expands our knowledge of phenotypes associated with gene mutations. We describe an 11-year-old female patient with a constellation of symptoms including congenital cataracts, gut dysmotility, sensory neuropathy, and bifrontal polymicrogyria. Whole exome sequencing was performed and identified a de novo heterozygous missense mutation in the ATPase motor domain of *cytoplasmic dynein heavy chain 1* (*DYNC1H1*), which is known to be involved in neuronal migration and retrograde axonal transport. The mutation was found to be highly damaging by multiple prediction programs. The residue is highly conserved, and reported mutations in this gene result in a variety of phenotypes similar to that of our patient. We report only the second case of congenital cataracts and the first of gut dysmotility in a patient with *DYNC1H1*, thus expanding the spectrum of disease seen in *DYNC1H1* dyneinopathies.

## 1. Introduction

*DYNC1H1* (OMIM #600112) encodes the heavy chain 1 of cytoplasmic dynein and controls microtubule binding as well as the recruitment of dynein components involved in retrograde axonal transport in neurons [1,2]. Mutations in *DYNC1H1* are associated with a wide spectrum of clinical manifestations including spinal muscular atrophy (SMA) [1,3,4,5], hereditary motor and sensory neuropathies [6], hereditary spastic paraplegia [7], malformations of cortical development (MCD), epilepsy, and intellectual disability [2,8,9]. Axonal defects are likely due to *DYNC1H1*’s role as the sole motor unit responsible for retrograde transport in axons [10]. Neuronal migration defects are likely due to *DYNC1H1*’s direct association with *LIS1* (*Lissencephaly 1*/platelet-activating factor acetylhydrolase isoform 1B, alpha subunit, *PAFAH1B1*) which is known to be critically required for normal neuronal migration during cortical development [9,11]. Reports of pediatric patients with *DYNC1H1* mutations have previously been published. The authors of those reports describe patients with a variety of symptoms including developmental delay, epilepsy, muscle weakness and wasting, microcephaly, spastic quadriplegia, foot deformities, and motor and sensory neuropathies [10]. Magnetic Resonance Imaging of those patients identified cortical malformations including pachygyria and polymicrogyria. Here we describe a patient with a *DYNC1H1* variant and symptoms not previously associated with this gene. Our observations expand the spectrum of manifestations of *DYNC1H1* mutations in humans.

## 2. Materials and Methods

We describe a patient under the care of physicians at Cincinnati Children’s Hospital Medical Center (CCHMC). All patient and parents were enrolled in a study upon informed written consent and assent as approved by the CCHMC Institutional Review Board (2014-3789, approved on 24 September 2015). Exome sequencing was performed at the CCHMC DNA Sequencing and Genotyping Core. In brief, genomic DNA was enriched with the NimbleGen EZ Exome V2 kit (Roche NimbleGen, Madison, WI, USA) and the exome library was sequenced using Illumina’s Hi Seq 2000 (Illumina, San Diego, CA, USA). Alignment and variant detection was performed using the Broad Institute’s web-based Genome Analysis Toolkit (GATK; [12]). Patient examinations were performed following best-practice guidelines and standard equipment.

## 3. Results

### 3.1. Patient Description

The proband is an 11-year-old female from a generally uncomplicated pregnancy and born full term via forceps assisted vaginal delivery secondary to cephalopelvic disproportion. Perinatally, she was noted to have hypotonia with poor weight gain and difficulty feeding. She initially required nasogastric feeds at seven months and, ultimately, gastric tube placement at one year of age. Extensive medical evaluation was significant for antroduodenal motility study performed at 17 months, demonstrated mild neuropathic changes in the stomach and small bowel, as well as post-prandial hypo-motility, consistent with pseudo-obstruction. She continued to have dysphagia, with choking and gagging, and chronic constipation. Upper GI, endoscopy, and swallow study did not indicate paralysis or weakness of pharyngeal muscles, dysmotility, or aspiration. Her constipation was managed with motility agents and laxatives, and she was gradually able to take more of her nutrition by mouth, although she continues to require some nutrition via her feeding tube. She was also found to have congenital cataracts, requiring bilateral implantation of intraocular lenses, as well as surgery to correct strabismus. A TORCH screen (*Toxoplasma gondii,* Other viruses, Rubella, Cytomegalovirus, and Herpes simplex) to assess infectious causes of congenital cataracts in utero was negative.

**Figure 1:**
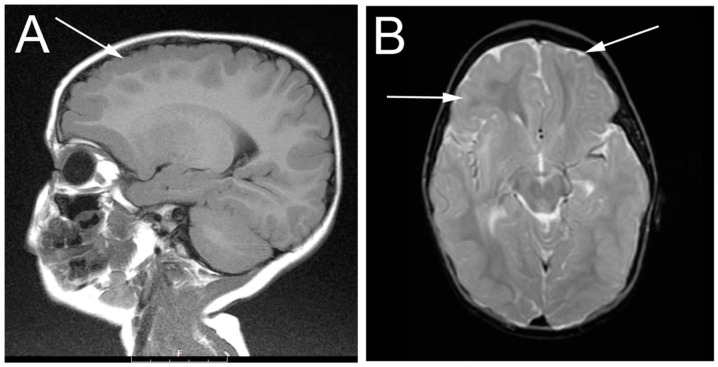
Magnetic resonance imaging (MRI) imaging of the patient at two years of age showing frontal lobe bilateral polymicrogyria (highlighted by arrows). (**A**) sagittal plane; (**B**) transverse plane.

Early behavioral intervention started at eight months. The girl became able to sit independently by two years of age, and walk at three years of age. She began to speak single words at age two, and simple sentences around age six. The patient presented for neurological evaluation at two years of age with concerns for seizures. As her routine EEG was normal, no antiseizure medication was prescribed. Muscle biopsy demonstrated neuropathic changes with large fiber groups consistent with denervation/re-innervation. Nerve conduction/EMG studies were not performed. Electroencephalogram (EEG) analysis at that time revealed diffuse bilateral epileptiform discharges with a bifrontal predominance. Magnetic resonance imaging (MRI) of the brain demonstrated symmetric bilateral polymicrogyria of the frontal lobe involving the superior and middle frontal gyri. There was hypo-myelination of subcortical white matter associated with the dysplastic areas. No other areas of polymicrogyria were seen and otherwise the brain, ventricles, and extra-axial spaces were normal in appearance (Figure 1).

She was lost to follow-up, but re-presented at age 10 with episodes of loss of consciousness, loss of tone, vomiting, and tachycardia lasting 5–10 s with post-ictal drowsiness. These episodes once again raised concerns that the patient was experiencing seizures. No dysmorphic features have been noted in the patient. There were no abnormal cardiac findings and no organomegaly. Neurologically, the patient exhibited cognitive and language impairment, with limited speech and cooperation on examination. Cognitive function was too low for formal IQ testing. She also exhibited impaired social skills, anxiety, and stereotypies. A motor exam revealed hypotonia, normal reflexes, and normal strength. Although the parents reported decreased sensitivity to extreme temperatures, sensory examination was not considered reliable due to her cognition.

### 3.2. Genetics

Given the constellation of symptoms combined with findings of polymicrogyria on MRI, we hypothesized a genetic etiology for the phenotypes seen in this patient. We performed next-generation exome sequencing on the patient as well as both parents. Non-synonymous coding variants were identified and compared to those found in control databases, including, NHLBI’s ESP6500 exome data [13], the 1000 Genomes Project [14], the EXAC project (Exome aggregation consortium, 2015) and an internal CCHMC sequencing control data control cohort. We analyzed the variant list for autosomal dominant de novo, homozygous recessive, and compound heterozygous mutations using three independent analysis platforms. All three analysis methods (Golden Helix Sample Variation Suite, Ingenuity Variant Analysis, and manual curation) identified the same likely candidate. The number of variants at each step for one of our analyses is shown (Table 1). Similar results were obtained from each.

**Figure 2:**
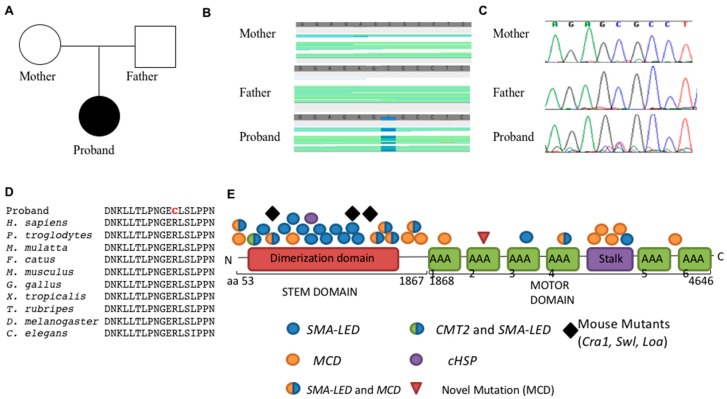
(**A**) Pedigree of family; (**B**) Whole exome sequencing identified a heterozygous C > T mutation in *DYNC1H1* confirmed by Sanger sequencing (**C**); (**D**) High conservation of the affected residue is shown through multiple species; (**E**) Summary of *DYNC1H1* mutations. (AAA: ATPase Associated with diverse cellular activities domain; *SMA-LED*: Spinal Muscular Atrophy-Lower Extremity Dominant; *MCD*: Malformation of Cortical Development; *CMT2*: Charcot-Marie-Tooth Type 2; *cHSP*: Hereditary Spastic Paraplegia; *Cra1*: *Cramping1*, *Swl*: *Sprawling*, *Loa*: *Legs-at-odd-angles*).

We identified a de novo autosomal mutation in *cytoplasmic dynein heavy chain 1* (*DYNC1H1*; c.6994C > T; p.R2332C; Figure 2C) in the proband. Sanger sequencing confirmed the mutation was present in the proband, but neither parent (Figure 2D). *DYNC1H1* is cytoplasmic dynein heavy chain and contains three major domains, the stalk that binds microtubules, the motor ATPase domain that provides the energy for motor function, and the stem domain that binds molecular cargo for transport. This de novo missense mutation in the second AAA ATPase subunit of the *DYNC1H1* motor domain was predicted to be deleterious by multiple bioinformatics prediction algorithms, including PolyPhen (1.0, “probably damaging” [15]) and MutationTaster (“disease-causing” [16]). In addition, the arginine residue at this position in *DYNC1H1* is highly conserved through nematodes (Figure 2D). The patient’s symptoms are consistent with a variety of the phenotypes observed in other patients and mice with mutations in *DYNC1H1* with the notable addition of congenital cataracts and gut dysmotility [10].

## 4. Discussion

The spectrum of disease caused by mutations in *DYNC1H1* has become evident with the advent of wide-spread genomic sequencing. We present here a patient with a mutation in the motor domain of *DYNC1H1* with a varied clinical presentation including failure to thrive, oral dysphagia, gut dysmotility, congenital cataracts, and developmental delay. MRI studies demonstrated bifrontal polymicrogyria. While the broad findings of developmental delay and polymicrogyria are consistent with previously reported patients with mutations in *DYNC1H1*, this report suggests other systems can be affected by dynein mutations. A very recent report also identified this mutation in a patient with many similar phenotypes, including congenital cataracts, microcephaly and failure to thrive [17]. Additionally, a patient with hereditary spastic paraplegia was found to develop bilateral cataracts in childhood [7]. As DYNC1H1 is expressed in the elongating fiber cells of the lens and possibly traffics vesicles along microtubules for reorganization of the fiber cell, it is possible that mutations in the motor domain disrupt the development of the lens and result in cataracts [18]. In addition, this is the first report of a *DYNC1H1* patient to display gut dysmotility with evidence of a mild enteric neuropathy. While the role of *DYNC1H1* in the enteric nervous system or neural crest cell migration into the gut is unknown, previously reported patients with mutations in *DYNC1H1* have exhibited peripheral motor and sensory neuropathies, which may be related to the proband’s phenotype [1]. Tissue-specific loss-of-function mutations in *DYNC1H1* will aide in determining the true requirement of *DYNC1H1* in the lens and enteric nervous system.

The mechanism underlying the wide range of clinical presentations in patients with *DYNC1H1* mutations has yet to be determined. Several mouse models with loss of *Dync1h1* function exist. The first homozygous knockout *Dync1h1* mouse died in early gestation (prior to embryonic day 8.5) due to abnormalities in the Golgi apparatus, endosome, lysosome, and massive defects in mitosis in the inner cell mass [19]. Later, ENU mutagenesis screens produced two independent point mutations in the stem domain of *Dync1h1*. *Legs-at-odd-angles* (*Loa*) and *Cramping1* (*Cra1*) heterozygous mice display a dominant motor weakness and sensory neuropathy phenotype characterized by a massive decrease in motor neurons (20%–50%) by gestational age E18.5 leading to paralysis and death within 24 h [20,21]. A subsequent analysis showed that *Loa* mutant mice also had neuronal migration defects in the cortex [22]. A radiation-induced knockout, *Sprawling* (*Swl*), contains a 9 bp deletion in the stem domain of *Dync1h1* resulting in a dominant phenotype with early onset sensory neuropathy and loss of muscle spindles in hind-limb muscles [23]. However, to date a mouse mutant with mutations in the *Dync1h1* motor domain has not been identified. Our patient’s phenotype is consistent with other patients with mutations in the motor domain, producing a largely MCD/epilepsy phenotype. As previously reported [1,24], patients with mutations in the stem domain are significantly more likely to develop motor and sensory neuropathies, while patients with mutations in the motor domain are more likely to develop malformations of cortical development and epilepsy, though these genotypes show some degree of overlapping phenotypes (Figure 2E). Scoto et al. recently highlighted the overlap in phenotypes of tail and motor domain mutations in a large cohort of SMA-LED patients with comorbid MCD [24]. As only the tail domain has been mutated in mouse models of a dyneinopathy, targeted mutations involving only the motor domain in mice will shed light on the role of each domain in the disease.

## Figures and Tables

**Table 1:** Exome Sequencing Analysis.

Familial Variant Analysis	# of Variants
Total Variants	114,284
Quality Control > 20 and Read Depth > 10	96,646
**De Novo Variant Analysis**	
Variants with MAF < 0.01 (Exac, 1000 genomes project, NHLBI ESP6500 exome data)	13,452
Coding, non-synonymous variants	1192
De novo mutations	70
Variants with alt allele > 0.3 freq. in proband	9
Variants supported by manual inspection of bam files	1 (*DYNC1H1*)
**Homozygous Recessive Analysis**	
Variants with MAF < 0.03 (Exac, 1000 genomes project, NHLBI ESP6500 exome data)	16,546
Coding, non-synonymous variants	1799
Homozygous recessive mutations	11
Variants supported by manual inspection of bam files	9
Remove variants seen in homozygotes in Exac	1
Gene causes human disease not seen in proband	1
**Compound Heterozygous Analysis**	
Variants with MAF < 0.03 (Exac, 1000 genomes project, NHLBI ESP6500 exome data)	16,546
Coding, non-synonymous variants	1799
Genes Represented with compound heterozygous mutations	16
Remove genes for which variants are seen as homozygotes in Exac	3
Gene known to causes human disease not seen in proband	2
Known gene expression not consistent with disease in proband	1

## Abbreviations

The following abbreviations are used in this manuscript:

CCHMC	Cincinnati Children’s Hospital Medical Center
*cHSP*	Hereditary Spastic Paraplegia
*CMT2*	Charcot-Marie-Tooth Type 2
*DYNC1H1*	cytoplasmic dynein heavy chain 1
EEG	Electroencephalogram
EXAC	Exome aggregation consortium
GATK	Genome Analysis Toolkit
MCD	malformations of cortical development
MRI	Magnetic Resonance Imaging
*PAFAH1B1*	platelet-activating factor acetylhydrolase isoform 1B, alpha subunit
*SMA*	spinal muscular atrophy
*SMA-LED*	Spinal Muscular Atrophy-Lower Extremity Dominant
TORCH	Toxoplasma gondii, Other viruses, Rubella, Cytomegalovirus, and Herpes simplex
